# Management of ASC-US/HPV positive post-menopausal woman

**DOI:** 10.1186/s12985-019-1145-5

**Published:** 2019-03-28

**Authors:** Maria Teresa Bruno, Angela Coco, Salvatore Di Pasqua, Giulia Bonanno

**Affiliations:** Department of General Surgery and Medical Surgery Specialities, Gynecological Clinic of the University of Catania, Policlinico, Via S. Sofia, 78 Catania, Italy

## Abstract

**Background:**

The aim of our study was to determine which diagnostic course is best to identify women at risk of CIN2+ among post-menopausal women with cytological diagnosis of ASCUS

**Methods:**

We selected women who had been post-menopausal for at least one year , and who had completed the entire diagnostic-therapeutic course that they had undertaken. The sample was divided into two arms: in the first arm, we considered 146 ASCUS positive women who had undergone the HPV test, colposcopy and then underwent more detailed diagnostics by means of LEEP or a scraping of the cervical canal. The second arm was made up of 124 ASCUS positive women who had undergone a vaginal administration of estriolo, the HPV test and colposcopy. Estriol was administered for 5 weeks: the first week one vaginal suppository every evening, the other four weeks the administration was twice a week. Then, the patients underwent colposcopy. In cases of positivity a biopsy was carried out, the patients positive for CIN2+ at the biopsy underwent excisional therapy using LEEP and were followed up. The patients, who were negative at colposcopy or with histological diagnosis of CIN1, were examined again at 1 year.

**Results:**

In the first arm the HPV test had an SE of 94%, an SP of 68%, NPV of 99%, and PPV of 28%. The PPV is very low because of the elevated percentage of false positives that the HPV test gave (71%). In the second arm the HPV test maintained its high SE (100%), an SP of 74%, a NPV of 100%, and a PPV of 43%. The use of estrogen increased the specificity of the test.

**Conclusion:**

It is important to say that the second arm indicates the use of local estrogen therapy only for ASCUS/HPV positive postmenopausal women. Therefore, the HPV test should be used as the first diagnostic possibility in cases of ASCUS in post-menopausal women, associating local estrogen therapy only with HPV positive women.

Epidemiology and basic research have confirmed that human papillomavirus (HPV) infection is a major cause of cervical intraepithelial neoplasia (CIN) and cervical cancer (SCC) [1], the latter being the fourth most common cause of death from cancer in women worldwide [2].

The persistence of the high-risk genotype of HPV (hr-HPV) is more likely to lead to the development of cervical cancer. Thus genotype determination [3] is fundamental as it identifies persistent hr-HPV infections and the women with HPV 16 infections (women at greater risk). In Italy the most prevalent genotypes are HPV 16, HPV 31 and HPV18 [4], whereas in the USA HPV 16, HPV 45 and HPV 51 are the most prevalent.

The prevalence of HPV infections is greater among young sexually active women, with an incidence curve below 25 years old, followed by a decline that is correlated with age. Recently, the Costa Rica Study and the Canadian Study have shown the presence of a second peak in the infection incidence curve in women over 55 years old. In the study on more than 8000 Costa Rican women, the prevalence of oncogenic HPV was 24.4, 15.4, 9.8, 9.7, 10.8 and 13.6% among women under 25 years old, 25–34 years old, 35–44 years old, 45–54 years old, 55–64 years old, and equal or greater at 65 years old, respectively [5] . It is not clear if the greater prevalence among older women found in this study was due to an increase in incidence, persistence or the re-activation of the infection previously acquired. In the Costa Rican cohort, the evaluation revealed that the increase observed in elderly women was due to persistent infection rather than new acquisition [5]. Another longitudinal study carried out at Guanacoste, in Costa Rica, also revealed that the acquisition of HPV diminished with age (*P* < 0.05) [6]. Changing sexual partner diminishes with the increase in age and, consequently, it is probable that also new infections decrease.

Previous studies reported that the risk of infection progression, prevalently to CIN3, is greater in elderly patients more than in younger ones [7–9], probably because of the fact that an infection detected in an elderly women is more probable to be persistent. If we look at the incidence for age for carcinoma of the uterine cervix reported in Germany [10] in 2013, the rate of raw incidence (RI) is higher in the groups aged 35–49 years and 50–64 years (16.5 per 100,000 and 14.8 per 100,000, respectively) and thereafter declined. Similar estimates have been reported in other populations in developed countries such as the UK and Australia [11, 12].

Elderly women are at higher risk of invasive cervical carcinoma due to the difficulty of early recognition of the significance of dysplastic anomalies that can appear. In particular, in postmenopausal women, due to the cellular alterations that are often dystrophic, thus also difficult to interpret, the diagnosis of ASC-US (atypical squamous cells - undetermined significance) is 2–3 times greater with respect to fertile age women [13]. Moreover, 73% of women with ASC-US who are over 40 years old are HPV DNA negative [14]. Moreover, because of hypoestrogenism, the Squamo-columnar Junction (SCJ) can often not be seen, thus making the colposcopic examination unsatisfactory.

ASC-US does not describe a true diagnostic entity, but includes a broad spectrum of alterations that can have an infective, phlogistic, reactive, metaplastic and also neoplastic pathogenesis. The true nature of ASC-US still has to be clarified, to identify women really at risk of developing CIN2 + .

The American Society for Colposcopy and Cervical Pathology, based on the ALTS (ASC-US - LSIL Triage Study) study, recommends an approach with the HPV test for those patients who have a diagnosis of ASC-US. This principle is also true for post-menopausal ASC-US positive women, because the Pap-test and colposcopy, the most common diagnostic methods, have a limited significance in menopause.

Moreover, the SICPCV (the Italian Society for Colposcopy and Vulvo-Cervical Pathology) states, “in post-menopausal women with ASC-US, without contraindications, it can be useful to repeat the Pap-test after vaginal treatment with estrogens” [15]. It is clear that the clinical picture of ASC-US in post-menopausal women is a diagnostic problem as it can produce an excess of false positives, the use of invasive examinations and strong emotive stress for women with an “uncertain” cytological presentation.

The aim of our study was to determine which diagnostic course is best to identify women at risk of CIN2+ among post-menopausal women with a cytological diagnosis of ASC-US.

## Materials and methods

We extrapolated from the colposcopy database of our department the data of 346 post-menopausal women who had been under our observation between 2012 and 2015 for cytological diagnosis of ASC-US for secondary screening. We selected women who had been post-menopausal for at least 1 year (age: 54.9 ± 5.0 years; time from menopause: 73.61 ± 53.2 months), and who had completed the entire diagnostic-therapeutic course that they had undertaken. Patients were excluded who had a history of carcinoma, chronic or autoimmune diseases and substitutive hormonal therapy or who had abandoned their diagnostic course.

The sample was divided into two arms:

In the first arm, we considered 146 ASC-US positive women who had undergone the HPV test. Colposcopy, which had been unsatisfactory in all the women because of the lack of vision of the entire transformation zone, encouraged us to submit the HPV positive women to a more detailed diagnosis with revision of the cervical canal in 12 cases and a LEEP in 45 cases. The cases that were negative at the HPV test were invited to repeat the Pap test after 1 year. Using the NPV (99%) of the HPV test we considered the HPV negative patients at low risk, and they underwent a cytological follow up; only one patient developed an HSIL lesion.

The second arm was made up of 124 ASC-US positive women who had undergone the HPV test. The positive HPV women had undergone a vaginal administration of Estriolo (E) administered for 5 weeks: the first week one vaginal suppository every evening, the other 4 weeks the administration was twice a week. Then, the patients underwent colposcopy. In cases of positivity (ATZ) a biopsy was carried out, the patients positive for CIN2+ at the biopsy underwent excisional therapy using LEEP and were followed up. The patients, who were negative at colposcopy or with histological diagnosis of CIN1, were examined again after 1 year.

The search for viral DNA (HPV-test) was carried out using PCR after the extraction of the cervical sample using Thin-prep. The automated DNA extraction was carried out on the NucliSenseasyMAG system (bioMérieux SA, Marcy l’Etoile, France) following the manufacturer’s HPV 1.1 protocol. Amplification of HPV DNA was accomplished by HPV-HS Bio (AB Analiticas.r.l, Padova, Italy) nested polymerase chain reaction (PCR) for the detection of HPV-DNA sequences within the L1 ORF, according to the manufacturer’s recommendations. HPV typing was carried out with specific probes for the most frequent HPV-types (HPV-type, AB Analitica s.r.l., Padova, Italy). HPV-typing allows the identification of 11 LR-genotypes (6, 11, 40, 42, 43, 44, 54, 61, 70, 72, 81) and 18 HR-genotypes (16, 18, 26, 31, 33, 35, 39, 45, 51, 52, 53, 56, 58, 59, 66, 68, 73, 82). Samples that were positive by nested-PCR but negative in reverse line blot for any of these types were considered as undetermined HPV. The cervical swab for the HPV test was taken from the endocervical canal and the transformation zone.

The Pap-test was carried out using conventional cytology. Cellular changes were classified according to the Bethesda classification system as negative, ASC-US, AGUS, LSIL, HSIL or indicating cancer. Histologic evaluation was performed with specimens collected by a colposcopy directed biopsy and/or cone specimens collected by the loop excision procedure. The histological slides were diagnosed according to the WHO classification as CIN2+ for all the cases of CIN2, CIN3 and SCC lesions. The women who showed a CIN2+ lesion underwent large loop excision of the transformation zone (LLETZ).

### Statistical analysis

We calculated the Sensitivity (SE), the Specificity (SP), the Positive Predictive Value (PPV) and the Negative Predictive Value (NPV) for the detection of CIN2+. We calculated significance (*P* < 0.5) with the Easy Fischer test.

## Results

The incidence of CIN2+ lesions in the two groups was 10.7% (37/346), almost all in HPV positive women, only one CIN2+ lesion was found in an HPV negative woman. The first arm had 57 HPV positive women (39%) and 89 HPV negative women (60.1%). All CIN2+ patients were HPV positive 28% (16/57), with the exception of one case (1.1%) that was HPV negative (1/89). We carried out 57 LEEP (57 HPV positive women) for a more detailed diagnosis, however, only in 28% of the cases (16 CIN2+ cases) was excision justified. The results are shown in Table 1.

**Table 1:** Diagnostic-therapeutic courses for the treatment of ASC-US positive post-menopausal woman in the two arms

	1st arm					2 th arm					
	women	Diagnosis LEEP	histology			women	Diagnosis E/colposcopy	Istology			LEEP
			negative	CIN2-	CIN2+			negative	CIN2-	CIN2+	
HPV test +	57 (39%)	57	4 (6,8%)	37 (63,7%)	16 (28%)	47 (37,9%)	47	12 (25,5%)	15 (31,9%)	20 (42,5%)	20
*HPV test -	89 (60,1%)	0				77 (62,1%)	77	70 (90,9%)	7 (9,1%)	0	0

*using the NPV (99%) of the HPV test we considered the HPV negative women of the first arm at low risk, and they underwent a cytological follow-up; only one patient developed a HSIL lesion

The second arm was made up of 124 women with cytological ASC-US who underwent the HPV test. The HPV positive women (47/124) were put on estrogen therapy. Colposcopy showed an abnormal transformation zone in 35 HPV positive cases that led to a targeted biopsy being carried out. The remaining cases underwent cytological follow-up. The results are shown in Table 1. There were 20 cases of CIN2+ (42.5%), adequately diagnosed and treated with LEEP (20 cases). Therefore, in this arm excision was justified in 100% of the cases.

In both groups, the cases of CIN2+ were treated with LEEP then follow-up. The accuracy values of the HPV test for CIN2+ in women with ASC-US of the two arms are shown in Table 1. In the first arm the HPV test had an SE of 94%, SP of 68%, NPV of 99%, and PPV of 28%. The PPV was very low because of the elevated percentage of false positives that the HPV test gave (71%). In the second arm the HPV test maintained its high SE (100%), an SP of 74%, NPV of 100%, and PPV of 43%. The use of estrogen increased the specificity of the test (*P* < .05).

## Discussion

The ASC-US/LSIL Triage Study (ALTS) is a clinical multicenter randomized study, sponsored by the National Cancer Institute, designed to compare three management options in ASC-US positive patients. The HPV-DNA test showed the most sensitivity and identified 96.3% (95% CI 91.6–98.8) of the women with CIN3+ [16]. This study, as confirmed by other similar studies, showed that the HPV DNA test for ASC-US had a sensitivity significatively greater for CIN2+ with respect to repeated cytology, thus colposcopy for women positive at the HPV test is more specific in older women where the prevalence of HPV is less and is also less expensive. Based on these results, the guide lines of both the American Society for Colposcopy and Cervical Pathology (ASCCP) and the European guide lines, recommend the HPV DNA test as a valid option for the management of ASC-US [17].

This is also true for post-menopausal ASC-US positive women, when both the Pap-test and colposcopy are not really helpful due to the state of hypoestrogenism that characterizes this period of life. The SICPCV (the Italian Society for Colposcopy and Vulvo-Cervical Pathology) states, “in post-menopausal women with ASC-US, without contraindications, it can be useful to repeat the Pap-test after the vaginal treatment with estrogens”. Vaginal estriol can be useful in increasing the accuracy of the Pap-test and in evidencing the SCJs in post-menopausal women.

In our group of post-menopausal women, we found an incidence of CIN2+ lesions of 10.7% (37 cases/ 346): the HPV test showed an increased sensitivity and, above all, an increased NPV. In fact, CIN2+ was found almost exclusively in the group of HPV positive patients, there was only one case in the group of HPV negative patients. In post-menopause ASC-US has a low positive predictive index for dysplastic lesions, while the HPV test in this age range maintains a high negative predictive index.

The TOMBOLA, multicentric clinical trial [18] results are in contrast with those of the ALTS study, not supporting the guide lines that recommend the triage of ASC-US woman positive at the HPV test because of the elevated sensitivity and the low specificity of the test. The problem becomes more serious in post-menopausal woman where the impossibility to see the transformation zone in ASC-US/HPV positive women necessitates a detailed diagnosis with excisional methods. Our results seem to support the hypothesis of the TOMBOLA Clinical Trial.

In the first arm the 57 HPV positive women, given the unsatisfactory colposcopy, underwent excisional therapy, however, this was adequate in only 28% of the cases (16 CIN2+). The 47 HPV positive women who underwent E therapy, that is colposcopy, resulted in an adequate therapy carrying out only 20 LEEP for the 20 cases of CIN2+ diagnosed.

For the women in the second arm the HPV test maintained its high SE (100%), an SP of 74%, NPV of 100%, and PPV of 43%. The use of estrogen increased the specificity of the test (*P* < 0.05).

These data indicate against a more aggressive management for ASC-US/HPV positive women, above all in post-menopause if not associated with local estrogen. The usefulness of treating post-menopausal women with local estrogen therapy concerns the fact that it increases the accuracy of the Pap-test and favors the visibility of the GSC thus avoiding ASC-US/HPV positive woman having to undergo a revision of the cervical canal or even a diagnostic LEEP.

These results indicate that the HPV test be used first to find women at risk and thus only ASC-US/HPV positive women should undergo local estrogen therapy.

## Conclusions

It is clear how the secondo arm (HPV test and estrogen) gave the best diagnostic-therapeutic course for the treatment of ASC-US positive post-menopausal woman. The HPV test identified women at risk, estrogen made the transformation zone visible making an accurate biopsy possible. Moreover, the advantage of not needing invasive investigations such as the revision the cervical canal or LEEP diagnostics should not be overlooked. It is important to say that the second arm indicates the use of local estrogen therapy only for ASC-US /HPV positive women. Therefore, the HPV test should be used as the first diagnostic possibility in cases of ASC-US in post-menopausal women, associating local estrogen therapy only with HPV positive women.
